# Evaluation of Non-Acid-Forming material layering for the prevention of acid mine drainage of pyrite and jarosite

**DOI:** 10.1016/j.heliyon.2020.e05590

**Published:** 2020-11-26

**Authors:** Muhammad Sonny Abfertiawan, Yoseph Palinggi, Marisa Handajani, Kris Pranoto, Ananda Atmaja

**Affiliations:** a Water and Wastewater Engineering Research Group, Faculty of Civil and Environmental Engineering, Bandung Institute of Technology, Indonesia; b Environmental Department, PT. Kaltim Prima Coal, Indonesia

**Keywords:** Acid mine drainage, Encapsulation, Pyrite, Jarosite, Soil science, Environmental engineering, Geochemistry, Environmental pollution, Environmental risk assessment, Environmental science

## Abstract

Encapsulation is a typical method used to prevent potential acid mine drainage (AMD) in overburden piles. In this method, Potentially Acid-Forming (PAF) material is covered with either Non-Acid-Forming (NAF) material or alkaline material to minimize water infiltration and/or oxygen diffusion through rock pores. The physical and chemical characteristics and thickness of the NAF material layer are critical factors affecting the successful prevention of AMD. Therefore, this study evaluated the method of NAF material layering using laboratory-scale column leaching tests. NAF layers with a ratio of 25 and 50% were used to cover PAF material containing pyrite and jarosite sourced from the Sangatta and Bengalon mining areas, East Kalimantan. The physical and chemical characteristics of leachate collected from samples watered on a weekly wet-dry cycle were analyzed by kinetic tests over a period of 23 weeks. The results showed a trend of increasing pH values and decreasing sulfate and metal concentrations in the leachate. This study shows that NAF layering is an effective method to prevent or minimize the generation of AMD.

## 1 Introduction

In 2016, Indonesia had considerable coal resources of 127 billion tons and coal reserves of 32.3 billion tons (Outlook Energy Indonesia, 2016). In 2018, Indonesia produced approximately 528 million tons of coal (Ministry of Energy and Mineral Resources, 2019). The region of Kalimantan holds the largest coal reserves, amounting to 14.9 billion tons, followed by Sumatra (11.2 billion tons) and Sulawesi (0.12 million tons). Abundant coal resources have the potential to have a multiplier effect, especially for the economic sector in mining area communities (Rosyid and Adachi, 2016; Singawinata, 2007). However, mining activities, especially open-pit coal mines, also have the potential to impact environmental ecosystems (Banerjee, 2014; Goswami, 2015; Johnson et al., 1987; Kaeser and Sharpe, 2001; Kimmel 1983), primarily through acid mine drainage (AMD). AMD is one of the major environmental problems in the coal and mineral mining industry (Devasahayam et al., 2017; Campaner et al., 2014). In open-pit mines, AMD forms by the release of sulfide minerals contained in the overburden or waste rock produced during excavation and backfilling activities. The sulfide minerals then react with oxygen in the air and with rainwater flowing on the ground surface or infiltrated into rocks with acid-forming potential. These reactions produce ferrous Fe, sulfate, and acidity (Singer and Stumm, 1970) as follows:

Reaction 1 $${2\text{FeS}}_{2\left(\text{s}\right)}+{15\text{O}}_{2\left(\text{g}\right)}+{2\text{H}}_{2}{\text{O}}_{\left(\text{l}\right)}\to {{2\text{Fe}}^{2+}}_{\left(\text{aq}\right)}+{{{4\text{SO}}_{4}}^{2-}}_{\left(\text{aq}\right)}+{{4\text{H}}^{+}}_{\left(\text{aq}\right)}$$

Reaction 2 $${{4\text{Fe}}^{2+}}_{\left(\text{aq}\right)}+{\text{O}}_{2\left(\text{g}\right)}+{{4\text{H}}^{+}}_{\left(\text{aq}\right)}\to {{4\text{Fe}}^{3+}}_{\left(\text{aq}\right)}+{2\text{H}}_{2}{\text{O}}_{\left(\text{l}\right)}$$

Reaction 3 $${{4\text{Fe}}^{3+}}_{\left(\text{aq}\right)}+{12\text{H}}_{2}{\text{O}}_{\left(\text{l}\right)}\to 4{\text{Fe}\left(\text{OH}\right)}_{3\left(\text{s}\right)}+{{12\text{H}}^{+}}_{\left(\text{aq}\right)}$$

Reaction 4 $${\text{FeS}}_{2\left(\text{aq}\right)}+{{14\text{Fe}}^{3+}}_{\left(\text{aq}\right)}+8{\text{H}}_{2}{\text{O}}_{\left(\text{l}\right)}\to {{15\text{Fe}}^{3+}}_{\left(\text{aq}\right)}+{{{2\text{SO}}_{4}}^{2-}}_{\left(\text{aq}\right)}+{{16\text{H}}^{+}}_{\left(\text{aq}\right)}$$

Ferrous iron can be oxidized by oxygen to form ferric iron; this reaction can be catalyzed by acidophilic bacteria (Reaction 2). Under certain environmental conditions, when the pH of water is less than 3.5, hydrolyzed ferrous iron will form iron hydroxide and 12 moles of acidity (Reaction 3). This reaction then forms golden-yellow iron hydroxide precipitates also known as "yellowboy." Under certain environmental conditions, ferric iron has the potential to oxidize the sulfide minerals again (Reaction 4). This reaction causes sulfate formation and greater acidity than oxidation reactions due to oxygen (Evangelou, 1995; Johnson and Hallberg, 2005). The oxidation reaction of sulfide minerals by ferric iron occurs rapidly and repeats until the ferric iron is depleted in the environment. Therefore, AMD that forms in the overburden or waste rock pile is difficult to resolve, requiring expensive chemical treatments (Kuyucak, 2002). AMD generation is a highly complex process influenced by several factors, including mineralogy, hydrology, geology, and local climate conditions (Abfertiawan et al., 2016; White et al., 1999). AMD has a low pH value of 2–4 (Abfertiawan et al., 2016; Bigham and Nordstom, 2000), which can lead to various other environmental problems, such as increasing concentrations of ${{\text{SO}}_{4}}^{2-}$ and metals, such as Fe^2+^, Al^3+^, and Mn^2+^ (Abfertiawan et al., 2016).

In open-pit coal mines, AMD has the potential to form in mining pit areas, as well as active overburden piles (Miller et al., 2019; Abfertiawan et al., 2011); this process can continue until the post-mining phase. Therefore, prevention efforts must be planned carefully to minimize AMD generation during the post-mining phase. A common AMD prevention method is the dry cover method using Non-Acid-Forming (NAF) material over Potentially Acid-Forming (PAF) material. This method is relatively widely used in Indonesian mines because of its efficiency and effectiveness (Matsumoto et al., 2017). NAF layering attempts to minimize oxygen diffusion and water infiltration into the overburden or waste rock disposal pile, thereby reducing the oxidation of sulfide minerals (Barton-Bridges and Robertson, 1989; RoyChowdhury et al., 2015; Skousen et al., 2000). Some studies have been conducted on the prevention of AMD generation using lime material (Zhou et al., 2017) or other alkaline materials, such as fly ash and bottom ash (Shimada et al., 2012; Win et al., 2020). The aim of this study is to evaluate AMD generation and the performance of preventive scenarios by conducting laboratory experiments using an NAF material layering over a period of 23 weeks. In addition, this study was also carried out to understand the rate of sulfide mineral oxidation and the behavior of AMD generation in various prevention scenarios. We utilized NAF material from a mining area to enhance its application efficiency. PAF material, containing two types of sulfide minerals, i.e., pyrite and jarosite, was used as the source of AMD in this study.

## 2 Materials and methods

### 2.1 Materials

**Figure 1:**
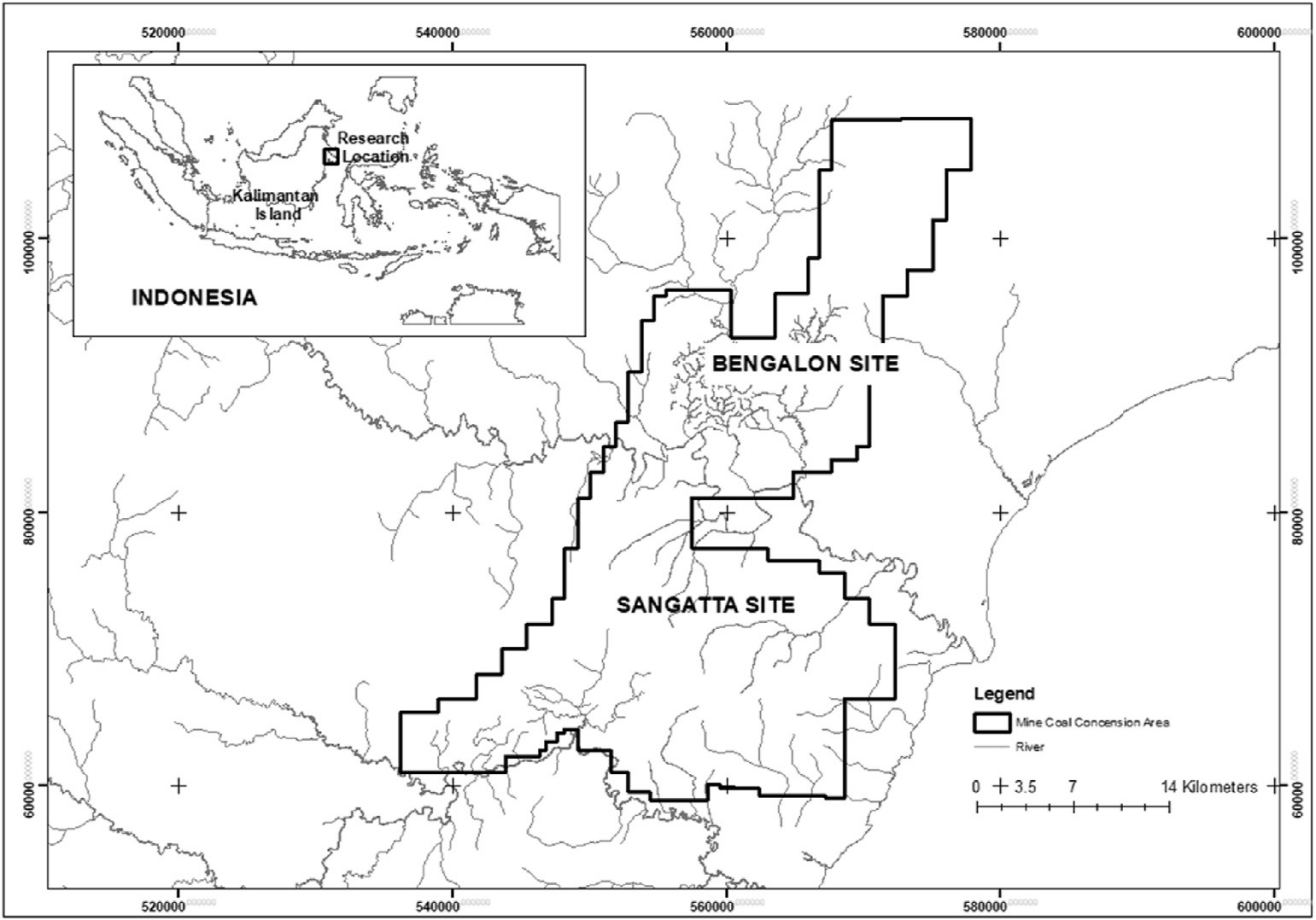
Location map of the study area.

Rock samples were taken from two mining areas at PT. Kaltim Prima Coal (PT. KPC), Sangatta and Bengalon, located in Kutai Timur District, East Kalimantan, Indonesia (see Figure 1). Two types of mudstone samples were collected, one with the potential to form acids, i.e., PAF material, and one that cannot form acids, i.e., NAF material. PAF samples were collected from the mining areas of Sangatta (S-02) and Bengalon (P-01), whereas NAF rocks were only collected from the Sangatta site (S-01). Samples were selected from different lithologies (see Table 1). All samples were crushed using a jaw crusher with ± 2-cm openings and filtered using a sieve with a 9.5-mm opening range.

**Table 1:** Lithology and characteristics of the samples.

No	Sample ID	Lithology	Characteristic	Location
1	S-01	Mudstone	NAF	Sangatta Site
2	S-02	Mudstone	PAF	Sangatta Site
3	P-01	Mudstone	PAF	Bengalon Site

### 2.2 Methods

Static tests were performed to determine the capacity for AMD generation; these included the paste pH, Total Sulfur, Acid-Base Account (ABA), and Net Acid Generation (NAG) tests. The tests were performed in accordance with the ARD Test Handbook, Amira International (2002). In addition, mineralogical analysis was conducted to observe the minerals within the rock samples. The analysis was conducted by X-Ray Diffraction (XRD), X-Ray Fluorescence (XRF), and Scanning Electron Microscopy with Energy Dispersive X-ray Spectroscopy (SEM/EDS) in accordance with standard procedures. These methods are widely used to both qualitatively and quantitatively analyze minerals in rock samples (Ritz and Klika, 2010).

**Figure 2:**
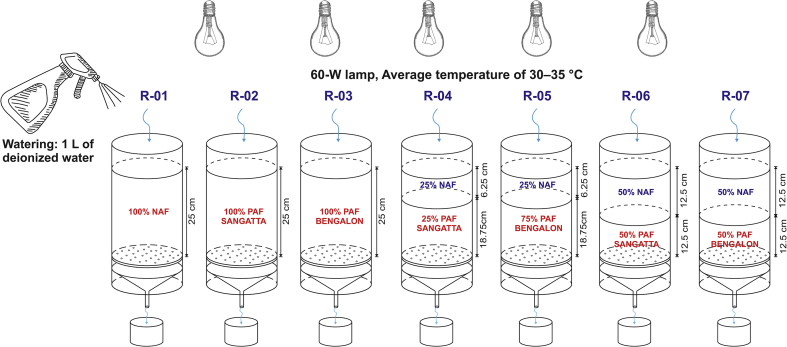
NAF Material Layering using laboratory-scale column leaching tests.

**Figure 3:**
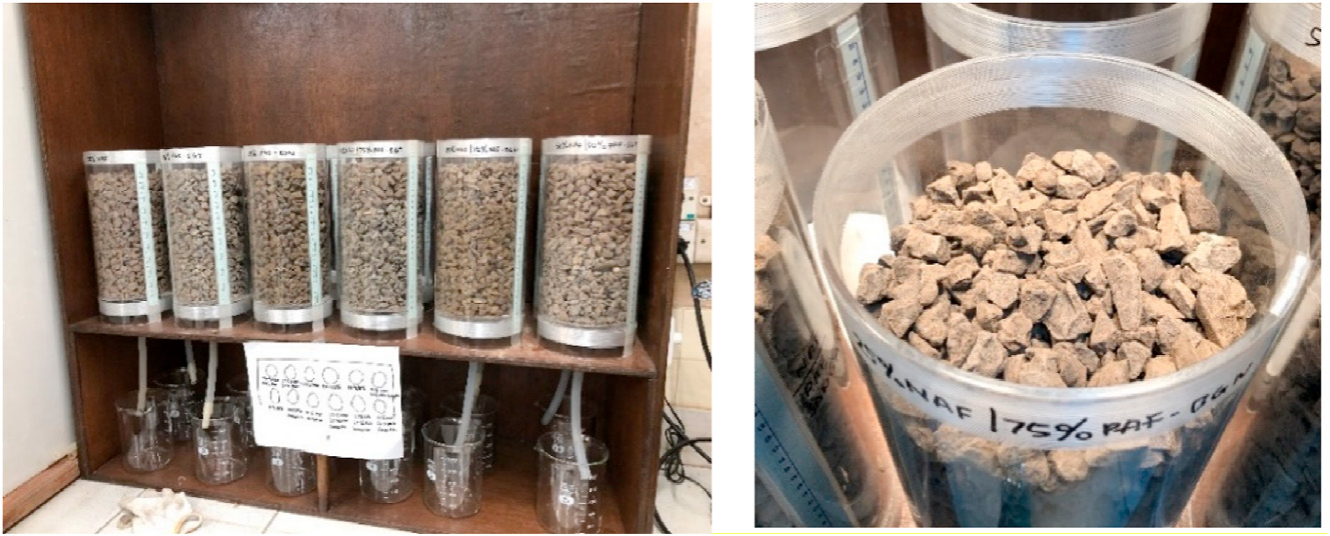
Column leach test (left) and the initial conditions of the sample on the surface (right).

Column leaching tests were conducted in the laboratory of PT. KPC. The column was composed of transparent acrylic material with a diameter of 15 cm and height of 30 cm (Figures 2 and 3). The height of the sample in the column from the bottom of the filter was 25 cm. A 60-W lamp was installed above the column to simulate sunlight or dry conditions with an average temperature of 30–35 °C. This temperature was employed to represent the conditions at the study area in East Kalimantan. Sample watering was conducted by adding 1 L of deionized water into each sample column on a weekly basis to simulate wet or rain cycles. Water infiltrated into the rock samples flowed out below the column and was collected for analysis. The rock leachate was analyzed according to its pH and concentrations of sulfate, total Fe, and total Mn. Water quality analysis was performed with reference to the Standard Methods of Analysis of Water and Waste from the American Public Health Association (APHA).

## 3 Results and discussion

### 3.1 Static tests: AMD identification

The static test results show that the S-01 sample had the highest acid neutralizing capacity (ANC) of 26.81 kg H_2_SO_4_/t. This value illustrates the amount of neutralizing material, including carbonate compounds in rocks, available to neutralize the formed acid. The ANC values of S-02 and P-01 samples were –36.07 and –7.24 kg H_2_SO_4_/t, respectively; a negative ANC indicates a significant acid mineral content in the rock samples. This high acidity value was also indicated by the low NAG pH of S-02 and P-01 (1.9 and 3.33, respectively). In addition, S-02 and P-01 had positive NAPP (Net Acid Producing Potential) values of 70.67 and 36,03 kg H_2_SO_4_/t, respectively. The NAPP illustrates the balance between the maximum potential acidity (MPA) and the ANC; a positive NAPP indicates the potential for acid formation.

Sample S-02 exhibited the highest concentration of total S at 1.13%. The method for total S analysis used in this study was considered quite conservative because it did not separate or distinguish different forms of S that may be contained in the samples, such as sulfides (pyrite or other sulfide minerals), sulfates, and native S. Sulfate and native S are not known sources of AMD. Table 2 lists the static test results revealing the characteristics of the three samples. Sample S-01 is categorized as NAF, whereas S-02 and P-01 are categorized as PAF. Sample S-02 sample has a greater acid formation potential than P-01, with MPA values reaching 34.60 kg H_2_SO_4_/t.

**Table 2:** Results of the static test.

No	Code	Total Sulfur (%)	MPA	ANC	ANC/MPA	NAPP	NAG pH	NAG		Category
								pH 4.5	pH 7	
1	S-01	0.54	16.53	26.81	1.62	–10.27	7.7	0	0	NAF
2	S-02	1.13	34.60	–36.07	–1.04	70.67	1.9	20.66	31.65	PAF
3	P-01	0.94	28.78	–7.24	–0.25	36.03	3.33	30.25	41.02	PAF

### 3.2 Mineralogy tests

**Figure 4:**
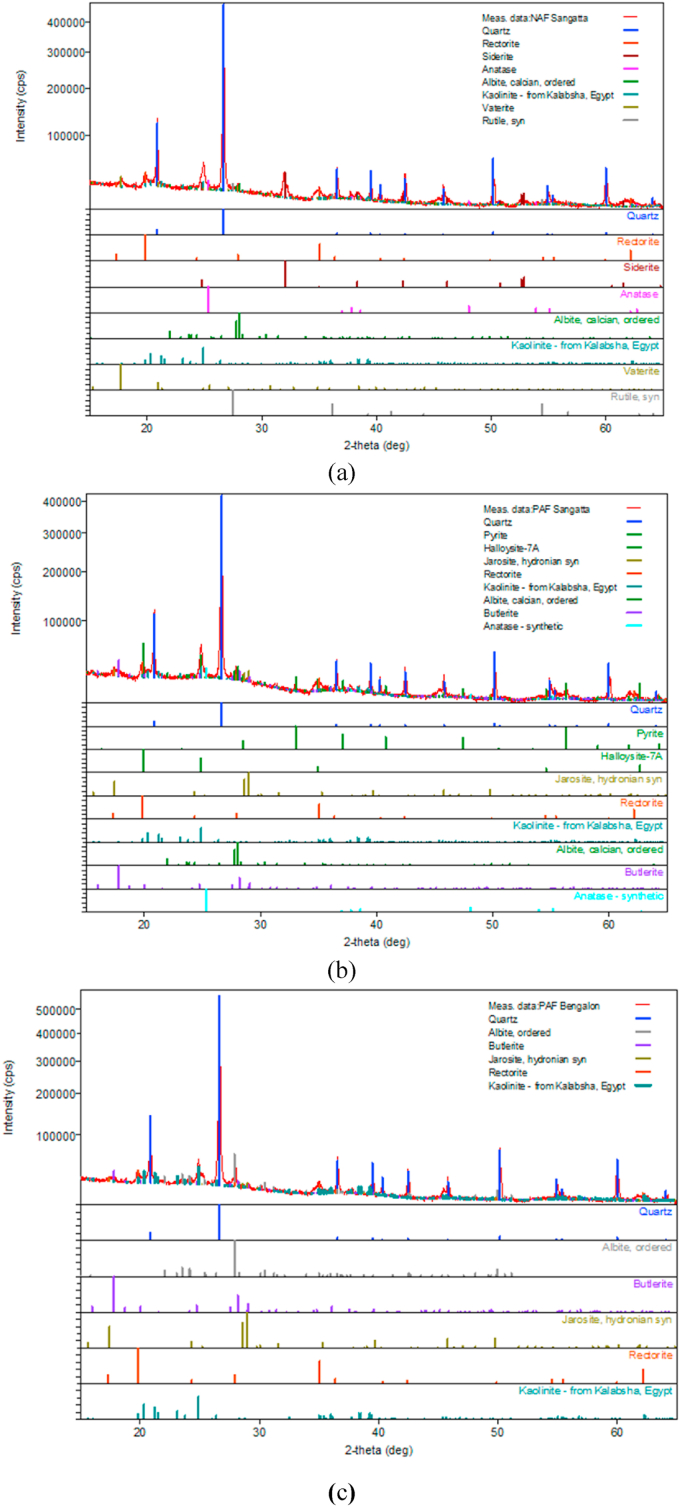
Results of the X-Ray diffraction analysis: (a) S-01, (b) S-02, and (c) P-01.

**Figure 5:**
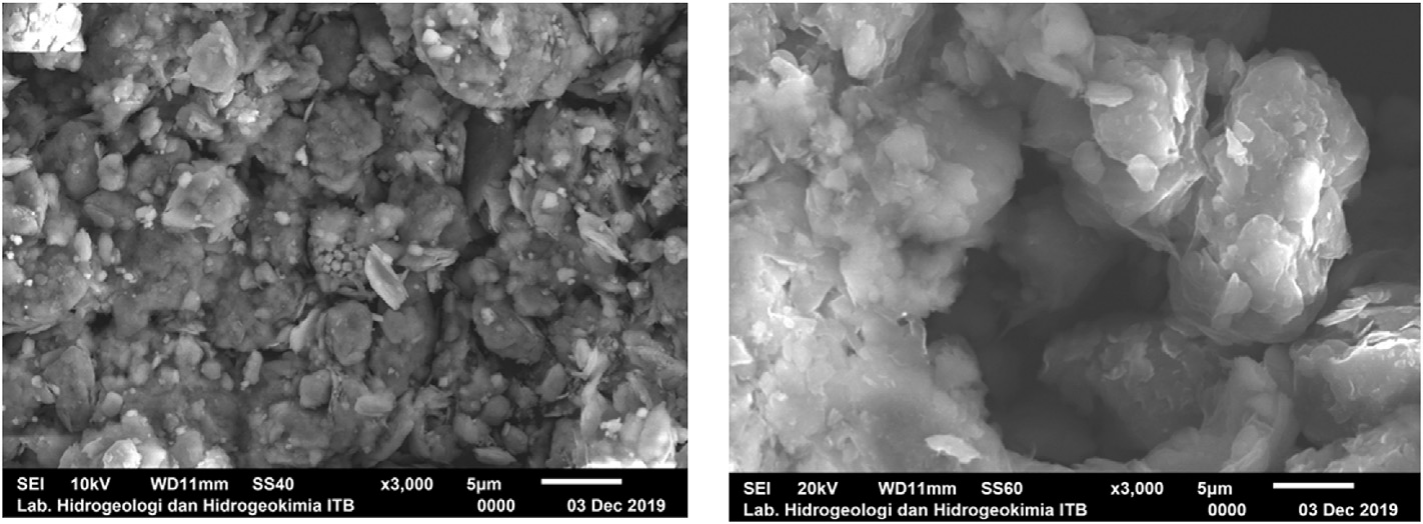
Pyrite (left) and jarosite (right) minerals from the SEM analysis.

Mineralogical tests were performed on all three samples to determine and confirm the types of sulfide minerals in each sample. Figures 4 and 5 show the XRD and SEM results, respectively. Sample S-01 can be classified as NAF because it did not contain sulfide minerals; however, it did contain carbonate minerals, such as vaterite (CaCO_3_) and siderite (FeCO_3_) (Figure 3a), both of which lead to high concentrations of ANC. Other minerals observed in sample S-01 were quartz (SiO_2_), rectorite ((Na,Ca)Al_4_((Si,Al)_8_O_2_0) (OH)_4_.2H_2_O), anatase [(TiO_2_), albite (Na,Ca)Al(Si,Al)_3_O_8_], kaolinite [(Al_2_Si_2_O_5_(OH)_4_], and rutile (TiO_2_).

Sample S-02 contained framboidal pyrite minerals as the main source of AMD generation (Figures 4b and 5-left). This framboidal morphology indicates that the pyrite mineral has the potential to have a greater oxidation rate due to its larger surface area. The other dominant minerals in sample S-02 were quartz [SiO_2_], jarosite [(K,H_3_O)Fe_3_(SO_4_)_2_ (OH)_6_], and halloysite [Al_2_Si_2_O_5_(OH)_4_]. Unlike sample S-02, sample P-01 did not contain pyrite minerals; jarosite [(K, H_3_O)Fe_3_ (SO_4_)_2_(OH)6] from the hydronium jarosite group was the only sulfide mineral observed in P-01 (Figures 4c and 5-right). Jarosite belongs to the iron-hydroxysulphate minerals and is often found in acidic and sulfate-rich environments, mining waste, and ore processing. Jarosite also has the potential to produce sulfate and H ions, thus forming AMD along river streams (Cogram, 2018).

### 3.3 Kinetic tests: characteristics of 100% column samples

**Figure 6:**
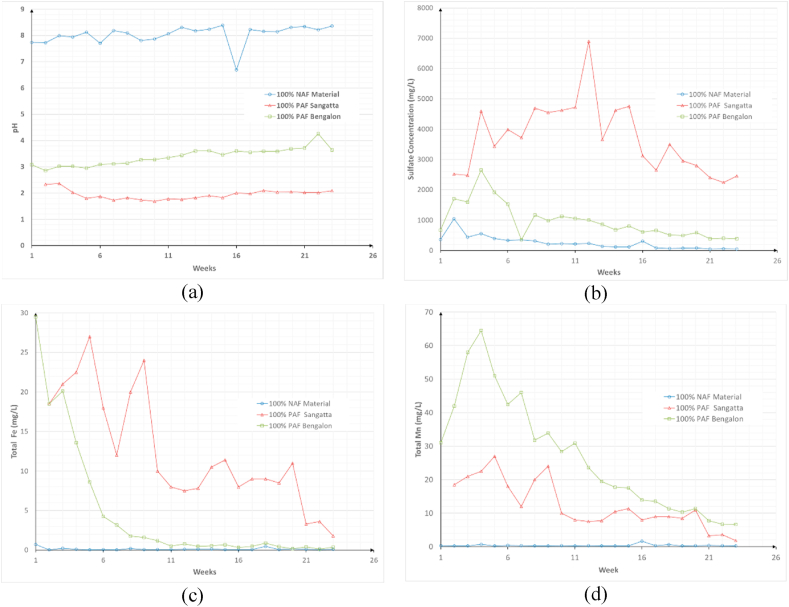
Characteristics of the leachate from 100% S-01, S-02, and P-01 columns: (a) pH value, (b) sulfate concentrations, (c) total Fe concentrations, and (d) total Mn concentrations.

Column leaching tests were conducted over a 23-week period. During this period, the leachate characteristics of different column conditions were analyzed to understand the behavior of AMD generation and the performance of the NAF material layering. The simulation results for columns containing 100% S-01, S-02, and P-01 material were consistent with the sample characteristics revealed by the static tests. The pH value of leachate from the S-01 sample column (NAF-Sangatta) ranged from 6–9 (see Figure 6a) and was typically above 8, which indicates the dominant presence of alkaline carbonate material. This was also illustrated by the static test results of sample S-01, which had a positive ANC value of 28.81 kg H_2_SO_4_/t and an NAG pH value of 7.7. This can be explained by the presence of vaterite (CaCO_3_) and siderite (FeCO_3_), i.e., carbonate minerals that contribute to the pH characteristics of the leachate. The low acid formation in S-01 was also indicated by the low sulfate concentration during the kinetic test, which ranged from 47–1,040 mg/L, with an average of 250.4 mg/L during the 23-week testing period (Figure 6b).

Sample S-02 from the Sangatta site had more acidic characteristics than sample P-01 from the Bengalon site; the leachate pH value was 1.5–2.5 and the concentrations of sulfate, which is a pyrite mineral oxidation product, reached 6,900 mg/L. The generation of AMD with pH < 4 leads to increased metal solubility; this was observed in the S-02 leachate, which exhibited the highest total Fe and Mn metal concentrations of 1,180 and 27 mg/L, respectively. This difference in the AMD formation potential of the two PAF samples was also indicated by the static test results.

The values of each parameter were lower in sample P-01 than in S-02. The jarosite present in P-01 had a relatively large influence on the lower rate of AMD formation than S-02. Jarosite is a secondary mineral that originates from the weathering and oxidation of pyrite and can be an important source of acidity in water (Park et al., 2013). According to Murray et al. (2014), jarosite minerals have the following general chemical equation: MFe_3_(TO_4_)_2_(OH)_6_, where M can have the form of Na, K, Ag, Tl, NH_4_, H_3_O, or ½Pb and TO_4_ can have the form of (SO_4_), (PO_4_), or (AsO_4_). This mineral is part of the alunite super group, which has a highly crystalline composition. This typically causes jarosite minerals to be stable and insoluble. In addition, jarosite plays an important role in overburden piles because of its ability to remove metals from solution during precipitation (Dold and Fontbote, 2001; Hudson-Edwards and Wright, 2011; Murray et al., 2014). These mineral characteristics may have led to the relatively non-acidic characteristics of the P-01 leachate when compared with the S-02 leachate. The concentrations of metals in sample P-01 were also lower, with total Fe and Mn concentrations of 0.13–29.5 and 6.6–64.5 mg/L, respectively. Moreover, these two parameters exhibited a significant decreasing trend (Figure 6c and d), whereas the pH of samples S-02 and P-01 increased over the 23 week period. This indicates that the oxidation of sulfide minerals began to diminish due to the decreasing mass of sulfide minerals. This was also suggested by the decreased concentrations of sulfate and metals in the leachate water.

### 3.4 Kinetic tests: 25 and 50% NAF layering

**Figure 7:**
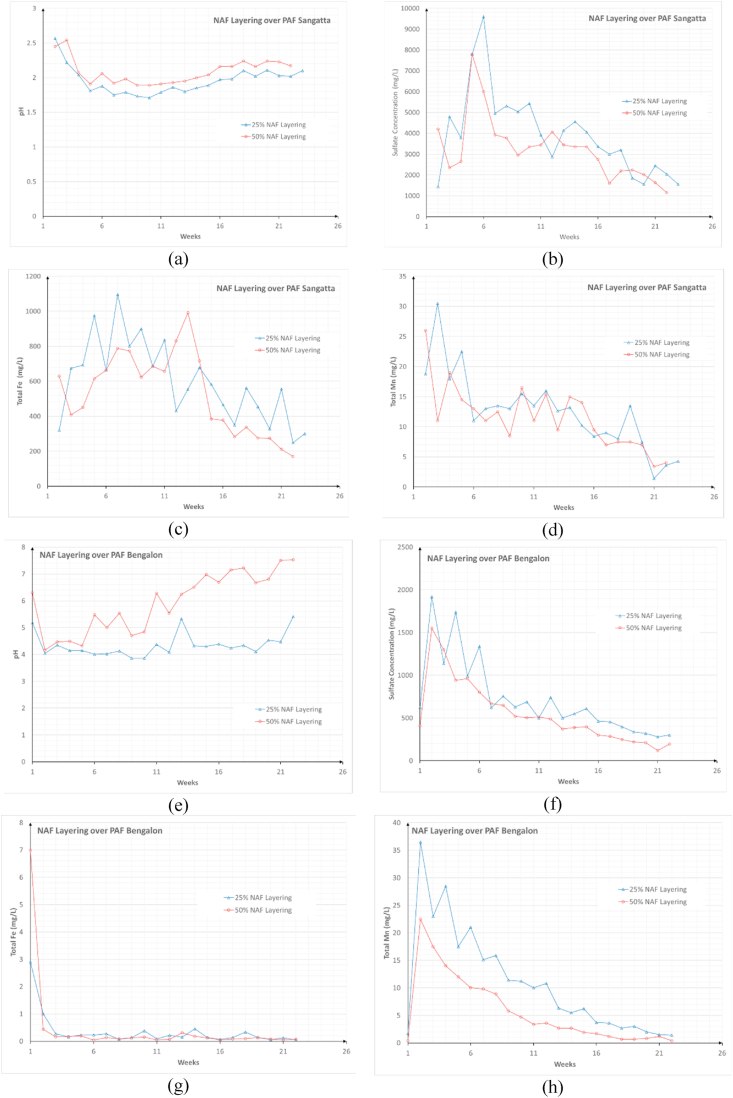
Characteristics of the leachate from the 25 and 50% of NAF material layering: (a) pH of the NAF layering over PAF Sangatta, (b) sulfate concentration of the NAF layering over PAF Sangatta, (c) total Fe concentration of the NAF layering over PAF Sangatta, (d) total Mn concentration of the NAF layering over PAF Sangatta, (e) pH of the NAF layering over PAF Bengalon, (f) sulfate concentration of the NAF layering over PAF Bengalon, (g) total Fe concentration of the NAF layering over PAF Bengalon, and (h) total Mn concentration of the NAF layering over PAF Bengalon.

Column leach tests were conducted with two layering scenarios, i.e., 25% NAF or 50% NAF, on two types of PAF material, i.e., PAF Sangatta (S-02) and Bengalon (P-01), over a 23-week period. The PAF Sangatta material, S-02, indicated a difference in performance between the layering scenarios of 25% NAF and 50% NAF, whereby the 50% NAF layering typically exhibited better characteristics. The pH values ranged from 1.89–2.54 for the 50% NAF layering and 1.71–2.57 for the 25% NAF layering (Figure 7a), which were higher than the pH of the 100% S-02 sample. This difference in acidity was also observed in the 25 and 50% sulfate concentration scenarios. The 25% scenario produced leachate with higher sulfate concentrations from 1,440–9,600 mg/L Figure 7b shows the fluctuations in the sulfate concentration between the two scenarios. Both scenarios exhibited increasing pH values up to the 23^rd^ week, followed by a decrease in the sulfate concentrations, total Fe, and total Mn (Figures 7b, 7c and 7d).

The kinetic test results for the 25 and 50% NAF layering scenarios on the Bengalon PAF material revealed notably different characteristics. This is a result of the characteristics of jarosite, which leads to the slow generation of AMD. The 50% NAF scenario had a higher pH value than the 25% scenario; the pH was above 4 and increased significantly throughout the 23 week period to reach a neutral pH. In contrast, the 25% scenario exhibited a slower increase in the pH toward a neutral value, only increasing from 4 to 5. This trend was also indicated by the decreasing concentrations of sulfate, total Fe, and total Mn. The presence of the relatively unreactive jarosite minerals notably reduced the concentrations of metals in the leachate water. Figure 6e–h shows the leachate behavior in the Bengalon PAF scenario. NAF layering simulations using both types of PAF material were performed to evaluate their performance and the factors controlling the prevention of AMD formation.

In general, encapsulation aims to minimize oxygen diffusion and/or infiltration of water into an overburden pile, thereby inhibiting the oxidation of sulfide minerals within the PAF layer (Pozo-Antonio et al., 2014; Gautama et al., 2013). NAF layering can also act as a neutralizer of AMD that has formed within the PAF layer (Matsumoto et al., 2018). This is achieved by the presence of alkaline materials, such as carbonates contained in the NAF material (Cravotta et al., 1990). Water flowing from the NAF layer carries high pH dissolved alkali material into the PAF layer. However, this method can only be applied if the NAF material has a sufficient buffering capacity, which can be indicated by a high ANC value (Wong et al., 1999) and the presence of carbonate minerals, as revealed by mineralogy tests. According to the kinetic test results of the 23-week period, the presence of carbonate minerals likely played a significant role in the NAF layering scenario in the kinetic tests. The NAF material had a high ANC value. The presence of carbonate vaterite (CaCO_3_) and siderite (FeCO_3_) material in the NAF samples inhibited the oxidation process of sulfide minerals, causing an increase in the pH value of the AMD that formed in the PAF layer. In addition, the oxygen diffusion factor into the PAF layer was not significantly retained by the NAF layer. In general, oxygen will begin to decrease at a depth of ~0.5 m from the surface (Doulati et al., 2010), depending on the physical characteristics of the material. According to Doulati et al. (2010), the pyrite oxidation rate has the potential to decrease significantly at lower depths of up to 1 m because of a rapid decrease in the oxygen concentration. In the kinetic test container, the degree of material consolidation also affected the changes in the concentration within the rock sample layer.

### 3.5 Rock weathering and metal precipitation: visual observations

**Figure 8:**
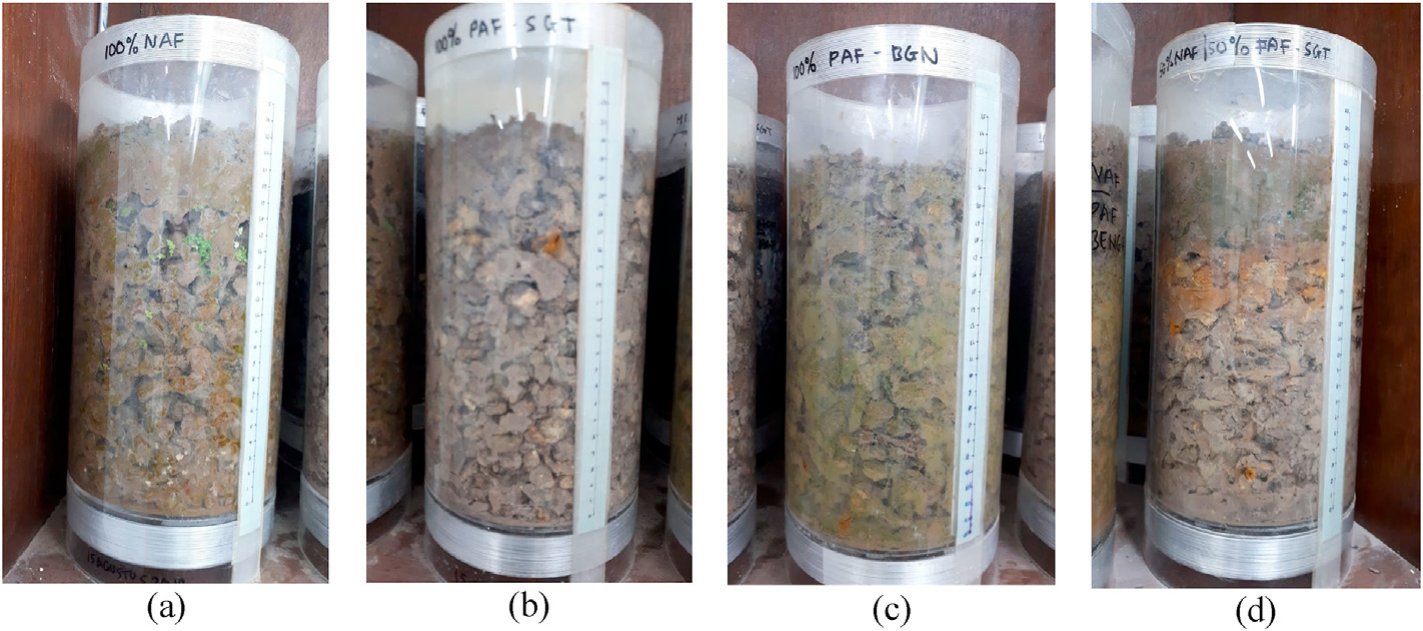
Physical conditions of the samples in the columns after 23 weeks of testing: (a) column with 100% NAF, (b) column with 100% PAF Sangatta, (c) column with 100% PAF Bengalon, and (d) column with 50% NAF layering.

Visual observations were also conducted during the kinetic tests to understand the physical changes in the column material. After 23 weeks of the test cycle, all samples decreased by 2–4 cm from an initial depth of 25 cm (Figure 8). This occurred because of rock weathering, which led to material consolidation. This phenomenon resulted in slower water infiltration during certain weeks. Material consolidation also has the potential to decrease the oxygen diffusivity due to reduced material porosity. However, in the column test case, the two variables, i.e., oxygen diffusion and water infiltration, did not significantly reduce the oxidation of sulfide minerals. The formation of metal precipitates was also observed in the column. Yellow-brown precipitates were observed in the PAF Sangatta layer (S-02), but not observed in the NAF layer (Figure 8a) or Bengalon PAF (Figure 8c),which contained jarosite minerals (Figure 8b and d). These precipitates were Fe(OH)_3_ that formed during the oxidation of pyrite minerals. In addition, dilution and carbonate from the NAF layer resulted in Fe^3+^ hydrolysis, which formed Fe(OH)_3_ precipitates (Dold, 2014). Iron (III) hydroxide is not stable and soluble when the environmental pH is less than 3.5 as the equilibrium will change and the Fe(OH)_3_ precipitates will form Fe^3+^ again. An equilibrium exists between aqueous Fe^3+^ and solid iron(III) hydroxide. According to the chemical reaction of AMD, the hydrolysis of Fe(OH)_3_ is the main acid producer (Williamson et al., 2006).

## 4 Conclusions

The results of this study prove that encapsulation using NAF material is a useful technique to prevent the formation of AMD in coal and mineral mines. Specifically, the use of NAF material increases the pH and reduces the sulfate and metal concentrations. The existence of carbonate material plays an important role in improving the quality of AMD prevention. At a larger scale, encapsulation with NAF material also minimizes oxygen diffusion and infiltration of water into the pile, especially when using PAF material. Further research should be conducted to determine the optimal encapsulation method to efficiently reduce oxygen diffusion and water infiltration. Jarosite minerals have the potential to produce acids; however, they are relatively unreactive when compared with pyrite minerals. According to the kinetic test results, samples containing jarosite minerals exhibited relatively better leachate characteristics than pyrite-bearing samples. Further analysis should be performed to determine the differences in the rate of oxidation of sulfide minerals during AMD generation. This study represents a preliminary step toward predicting the potential of AMD formation.

## Declarations

### Author contribution statement

Muhammad Sonny Abfertiawan: Conceived and designed the experiments; Performed the experiments; Analyzed and interpreted the data; Contributed reagents, materials, analysis tools or data; Wrote the paper.

Yoseph Palinggi: Performed the experiments; Analyzed and interpreted the data.

Marisa Handajani, Kris Pranoto: Analyzed and interpreted the data.

Ananda Atmaja: Performed the experiments.

### Funding statement

This work was supported by the 10.13039/501100015689Bandung Institute of Technology through the Water and Wastewater Engineering Research Group (KK-RALC) at the Faculty of Civil and Environmental Engineering from a research grant as part of the program titled, "Program Penelitian, Pengabdian kepada Masyarakat, dan Inovasi (P3MI) Kelompok Keahlian ITB 2019." The Environment Department of PT. Kaltim Prima Coal and PT. Ganeca Environmental Services also provided financial and technical support for this study.

### Data availability statement

Data included in article/supplementary material/referenced in article.

### Declaration of interests statement

The authors declare no conflict of interest.

### Additional information

No additional information is available for this paper.
